# Photodoping of metal oxide nanocrystals for multi-charge accumulation and light-driven energy storage

**DOI:** 10.1039/d0nr09163d

**Published:** 2021-05-07

**Authors:** Michele Ghini, Nicola Curreli, Andrea Camellini, Mengjiao Wang, Aswin Asaithambi, Ilka Kriegel

**Affiliations:** Department of Nanochemistry, Istituto Italiano di Tecnologia (IIT) via Morego 30 16163 Genova Italy; Dipartimento di Chimica e Chimica Industriale, Università degli Studi di Genova Via Dodecaneso 31 16146 Genova Italy; Functional Nanosystems, Istituto Italiano di Tecnologia (IIT) via Morego 30 16163 Genova Italy ilka.kriegel@iit.it

## Abstract

The growing demand for self-powered devices has led to the study of novel energy storage solutions that exploit green energies whilst ensuring self-sufficiency. In this context, doped metal oxide nanocrystals (MO NCs) are interesting nanosized candidates with the potential to unify solar energy conversion and storage into one set of materials. In this review, we aim to present recent and important developments of doped MO NCs for light-driven multi-charge accumulation (*i.e.*, photodoping) and solar energy storage. We will discuss the general concept of photodoping, the spectroscopic and theoretical tools to determine the charging process, together with unresolved open questions. We conclude the review by highlighting possible device architectures based on doped MO NCs that are expected to considerably impact the field of energy storage by combining in a unique way the conversion and storage of solar power and opening the path towards competitive and novel light-driven energy storage solutions.

## Abstract 2

Light-driven multi-charge accumulation (*i.e.*, photodoping) of doped metal oxide nanocrystals opens the way to innovative solutions for the direct conversion and storage of the solar energy.

## Graphical abstract

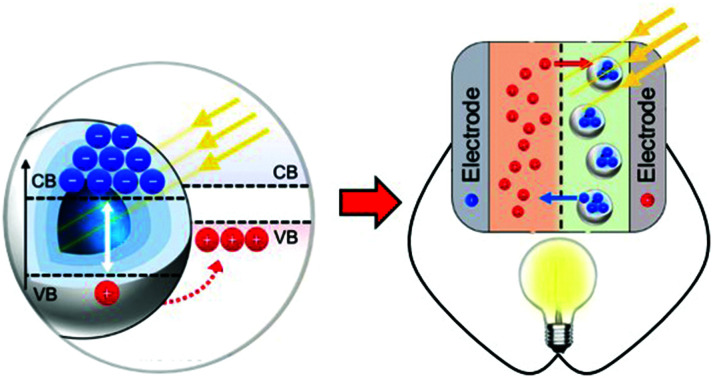

## Introduction

The global energy crisis together with the growing demand for powered devices represents one of the main challenges of today's society.^1^ Consequently, the realization of devices with ever-smaller dimensions and the reduction of cost and power consumption of the electronic parts are essential requirements for the development of innovative and sustainable technologies.^2^ A fundamental prerequisite for novel powered devices is their energy autonomy over extended periods of time. Power generation based on energy harvesting from clean renewable sources (*i.e.*, light, wind, mechanical vibrations, or temperature changes) requires their combination with an energy storage unit to overcome the fluctuations related to their intermittent availability.^3^ Light-powered energy storage solutions have become a promising direction.^4^ In such devices, often energy conversion and storage are carried out in stand-alone units each covering a separate functionality. The active materials involved pursue independent tasks: the process of solar energy absorption followed by charge separation is distinct from the charge accumulation.^5^ Loss mechanisms related to the coupling of the two subunits often limit the overall efficiency. Moreover, single (or even less) charge transfer reactions are involved.^5^

Doped metal oxide nanocrystals (MO NCs) are potential candidates combining light conversion and storage in the same material.^6^ As a consequence, doped MO NCs have been studied as alternative solution-processable materials for many different applications over the past years.^7–10^ Particularly interesting is the opportunity to introduce multiple free charge carriers into the MO NC *via* light absorption. This light-induced doping process, in the following termed photodoping, offers a convenient way to *in situ* and non-destructively modulate carrier densities in NCs.^11^ The photo-induced n-type doping of NCs is accompanied by the capture of photogenerated holes with suitable hole quenchers. This results in the accumulation of extra electrons within the NCs, while charge compensating cations deposit on the NC surface.^11^ This method has been successfully applied to a number of semiconductor NCs. For example, photodoping was implemented to generate and manipulate electron spins in magnetic semiconductor NCs for investigation of carrier–dopant interactions.^12^ In Cd-based chalcogenide NCs, such as CdSe NCs, photodoping was used to increase the level of doping involving the occupation of quantized energy levels.^11^ Photochemically n-doped colloidal CdSe NCs were implemented for manipulating hot electron dynamics.^13^ However, Cd-chalcogenides possess shallow valence bands and consequently very reactive hole quenchers are required to perform efficient photodoping.^11^ Moreover, only a very limited number of electrons (*e.g.*, two conduction-band electrons per NC) are introduced with photodoping, owing to the discrete energy levels involved in the process.^11^ Notably, MO NCs are able to store multiple delocalized charges per NC after light absorption reaching tens^14,15^ to hundreds^6,16^ of extra conduction band electrons per NCs. The resulting nanoscale ‘supercapacitors’ display attractive capacitance values competing well with materials currently employed in state-of-the-art energy storage systems with the added value of being charged by light.^17^ Recently, this solution-based photodoping process, has been transferred to an all-solid-state platform.^18^ Despite these promising results, up to now in MO NCs only light-driven charge accumulation was demonstrated, while the fully-working device and the demonstration of light-driven energy storage remains an open task.

In this review, we introduce the light-driven charging of doped MO NCs by discussing the most important physical and chemical processes involved, the theoretical background, and open issues. We highlight scenarios envisaged to exploit the photodoping and multi-charge accumulation processes of this emergent material for energy storage solutions, taking a step towards the direct conversion of solar radiation into readily available electrical energy.

## Photodoping and multi-charge accumulation of doped MO NCs

**Fig. 1:**
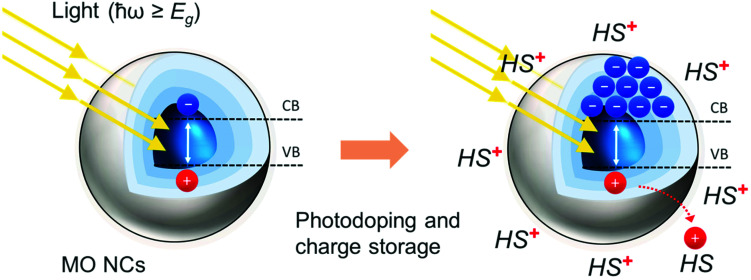
Photodoping and multiple charge accumulation in doped MO NCs. Light absorption with energy (*ħω*) above the bandgap (*E*_g_) promotes an electron from the valence band (VB) to the conduction band (CB). By removing the photo-generated hole with a hole-scavenger (HS), the electron–hole pair recombination is suppressed and the extra electron is stored in the nanocrystal. The absorption of several photons results in the accumulation of multiple photoelectrons in the NC and leads to the overall photodoping of the material.

Doped MO NCs display unique optical properties determined by their free carrier density in the range of 10^20^–10^21^ cm^−3^. The free carrier density is controlled by the level of doping, ultimately delivering a tool to continuously tune the optical properties in a broad range of wavelengths. Metal oxides NCs present a rich variety of synthesis methods ranging from solution based, such as solvothermal methods, to sol–gel methods, to microemulsion methods, to microwave-assisted methods, to solvothermal methods, to vapor state based and biological methods.^19,20^ Developments in non-aqueous sol–gel methods managed to overcome many drawbacks of aqueous systems by exploiting inert organic solvents^21,22^ and recently several doped MO NCs (such as ITO NCs) have been synthesized using a continuous growth approach with slow-injection of precursors, significantly improving the control over the main parameters of the synthesis.^23,24^ Doping at the stage of synthesis exploits several chemical strategies, including (1) intrinsic doping due to lattice point vacancies (such as oxygen vacancies that introduce two additional electrons), (2) extrinsic interstitial doping (such as Cs doping in WO_3_ NCs), or (3) aliovalent substitutional doping with donor impurities (such as Sn for In_2_O_3_ or Fe dopants for ZnO).^17,25–28^ Post-synthetic doping and dynamic modulation of the carrier density relies on the introduction of extra charges in a capacitive charging process by applying either an electrochemical potential or by interaction with light beyond their bandgap.^29,30^ In the photodoping process (Fig. 1), the absorption of photons with energy beyond the bandgap of the MO NCs promotes the accumulation of extra charges in the NC. The quick removal of the extra holes by a sacrificial hole scavenger at the nanocrystal surface suppresses electron–hole pair recombination (radiative and non-radiative). This stabilizes quasi-permanently the extra electrons (in inert environments), leading to a variation of the carrier density of the material.^17,31,32^ Initially, Haase *et al.* demonstrated photodoping on ZnO NCs.^31^ Since then, photodoping has been extended to other MO NCs such as tin-doped In_2_O_3_ (ITO).^32–34^ The participation of hole scavengers in the photodoping process is crucial. The hole capture process (<15 ps) is an order of magnitude faster than the competing Auger recombination (∼150 ps) of the photogenerated electron–hole pair, making the accumulation of multiple conduction-band electrons possible.^16^ Multicarrier Auger recombination rates increase with the number of excess charge carriers and compete kinetically with the hole scavenger's photo-oxidation. The equilibrium of these two processes ultimately results in an average maximum number of excess electrons that can be stored per NC. The photodoping process, thus, depends strongly on the nature of the hole scavenger and is not necessarily an intrinsic property of the NCs themselves.^16^ In fact, the average maximum number of stored charges per single NC varies significantly when different hole scavenger molecules, such as alcohols, organic acids, hydrocarbons, or even organometallic compounds are employed.^16^ Strong hole scavengers, *e.g.* Li[Et_3_BH] and K[Et_3_BH], lead to a significantly greater number of stored charges, with the drawback of making the photodoping process irreversible.^35,36^ The light-induced oxidation of hole scavengers at the surface of the MO nanoparticles, occurs either through direct oxidation or indirectly by reacting with interfacial OH radicals resulting from the trapping of the holes at –OH surface groups.^14,16,36–38^ The positively charged hole scavenger molecule is electrostatically attracted to the surface of the NC, guaranteeing the overall neutrality of the system. The identity of the charge-compensating counteraction induces systematic shifts in the Fermi level (*E*_F_), having important implications on the electronic properties of the NC.^39^ For example, in the case of ZnO nanocrystals, the charge storage equilibrium is mainly determined by the ethanol (EtOH) oxidation process, where two EtOH molecules accept extra holes from the NC, resulting in the formation of acetaldehyde (ZnO + ½CH_3_CH_2_OH → ZnO^−^ + H^+^ + ½CH_3_CHO).^14^ The maximum storage in ZnO NCs is thermodynamically defined by the reverse process, in which acetaldehyde is hydrogenated, hindering the accumulation of additional electrons (ZnO^−^ + H^+^ + ½CH_3_CHO → ZnO + ½CH_3_CH_2_OH). Acetaldehyde accumulation and proton-driven surface reactions induce Fermi level pinning, deforming the electronic structure of the nanocrystal.^14^

Besides the hole scavenger, the photodoping process is controlled by several factors.^14,32^ The maximum number of extra electrons that can be introduced *via* photodoping is independent of the initial carrier density of the doped MO NCs. Several works reported that the final carrier density that can be reached through photodoping (when using the same hole scavenger) is a constant value.^16,32^ Consequently, the total amount of photo-accumulated charges displays a cubic growth radius law (*R*^3^) with the volume of the NC.^16^ Moreover, Coulomb interactions and surface reactions can also influence the photodoping processes.^16,40^

## Determining the capacitance of photodoped MO NCs

**Fig. 2:**
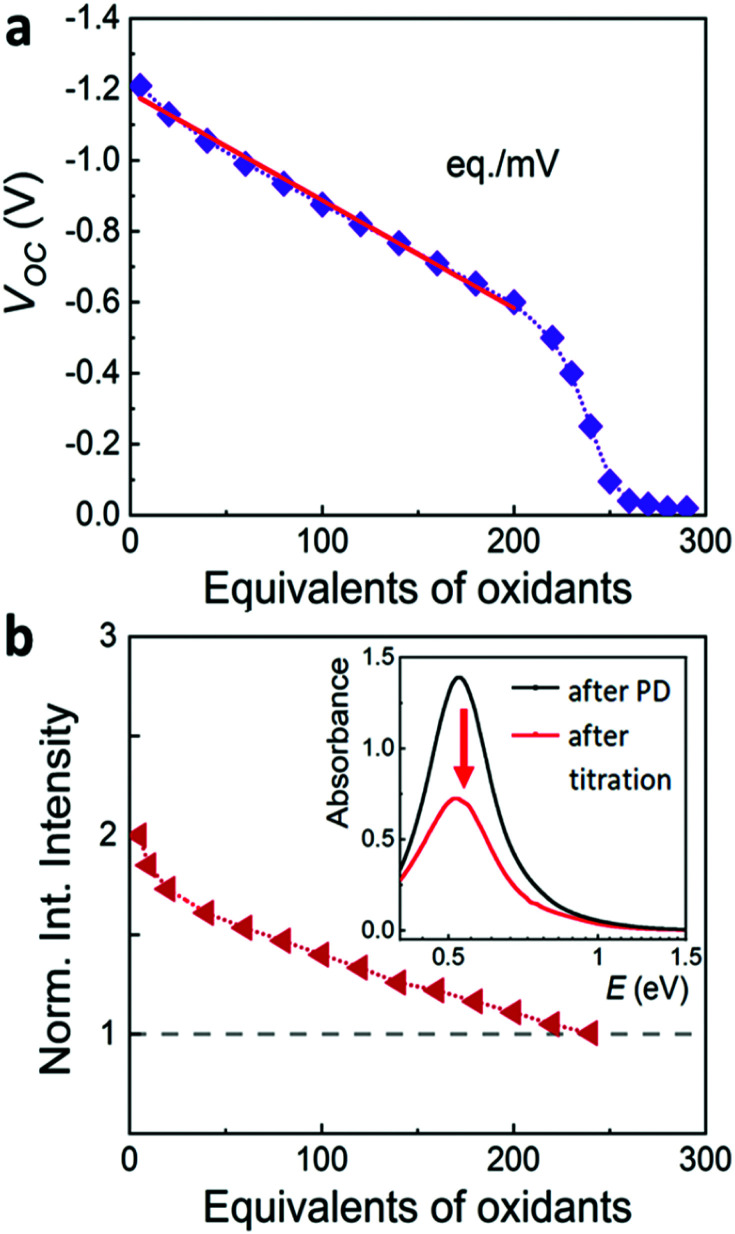
(a) Illustration of a typical monitoring of the open circuit voltage (*V*) in an electrochemical cell upon potentiometric titration by introducing oxidants in the solution. From the slope of this plot it is possible to extract capacitance values with *C* = Δ*q*/Δ*V*, where the number of charges *q* is determined by the oxidant added. (b) Illustration of a typical normalized integrated intensity of the plasmonic resonance (LSPR) as a function of the equivalents of oxidants added until full recovery of the initial optical properties. Inset shows the decrease in the intensity of the plasmonic resonance upon addition of increasing equivalents of oxidants (red arrow).^32^

Potentiometric titration is a powerful method for measuring the Fermi level and the capacitance of photodoped MO NCs (Fig. 2).^14,15,41^ Briefly, a precise concentration of titrant (molecular oxidants) is added to the suspension of photodoped nanocrystals. This triggers the oxidation reaction of the NC with the extra photoelectrons and the subsequent recovery of the initial conditions.^32^ Under galvanostatic conditions (no current flowing), the working electrode and the colloidal dispersion can be kept constantly at the same potential with the support of a potentiostat. In this case, it is possible to extract the Fermi level (*E*_F_) of the NCs and follow variations in its values by tracking changes in the half-cell potential.^16,40^ The analysis of the slope of the open-circuit potential (*V*_OC_, between the working and counter electrodes) *versus* the added equivalents of titrant allows extracting the capacitance *C*, calculated as *C* = Δ*q*/Δ*V*, where Δ*q* is the change of the charge carriers, and Δ*V* is the corresponding change in electrochemical potential (see Fig. 2a).^17^

The accumulation of electrostatically attracted positive charges on the surface of the negatively photodoped MO NCs resembles an electric double-layer. This indicates that the charged NCs can be understood as soluble nano-sized supercapacitors.^17,39^ Indeed, signatures of capacitive and pseudocapacitive charging dynamics have been reported in various MO NCs.^17,18,29,39,42^ Unlike standard capacitors, supercapacitors take advantage of the double-layer electrostatic capacitance and electrochemical pseudo-capacitance,^43^ resulting in high power combined with high energy storage capacity.^44,45^ Electric double-layer capacitors (EDLC) operate on principles similar to those of conventional electrostatic capacitors. Instead of using two conductive materials separated by a dielectric layer to store energy, EDLCs store electrical charges accumulated at the interface between the surface of a conductor and an electrolytic solution, where the phenomenon of the double-layer effect occurs.^46^ This purely electrostatic effect stores electrical charges as in a conventional capacitor, forming a static electric field, which corresponds to the intensity of the applied voltage. The bilayer roughly acts as a dielectric layer in a conventional capacitor.^47–49^ The charge separation is around a few angstrom^50^ and implemented in the following equation for the double-layer capacitance (*C*_dl_):^39^1
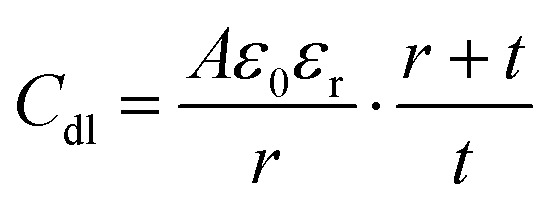
with *A*, *ε*_0_, *ε*_r_, and *r* referring to the sphere's surface area, the permittivity of free space, the dielectric constant of the solvent, and the nanocrystal radius, respectively. This equation is the extension of the classical electrostatic capacitor (
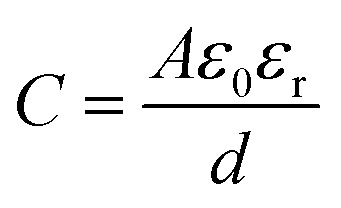
, with *d* being the separation of the plates) by an additional term that accounts for the thickness *t* of the electrical double layer.^39^ Photodoping of MO NCs, however, involves charge transfer reactions across the solid–liquid interface, which resembles more closely to the phenomenon of pseudo-capacitance. The pseudo-capacitance stores electrical energy through reversible faradaic redox reactions on the surface, accompanied by an electron charge-transfer.^51^ Usually a very fast sequence of reversible redox, intercalation, or electrosorption processes accompany such faradaic charge transfer, while no chemical reaction occurs.^51,52^ Such processes however are still under investigation, indicating that the real phenomenon is still an open issue. A more detailed study is required to support properly the characterization of devices based on photodoping. Careful control over surface chemistry, including surface ligands, will allow determining key properties of charge transfer across the interface. This will provide a comprehensive understanding of both the thermodynamics of charge transfer at the nanoscale and the charge storage mechanism (*e.g.*, pseudocapacitive *vs.* double-layer capacitive) in the nanocrystals.

## Spectroscopic and theoretic approaches to photodoping of MO NCs

**Fig. 3:**
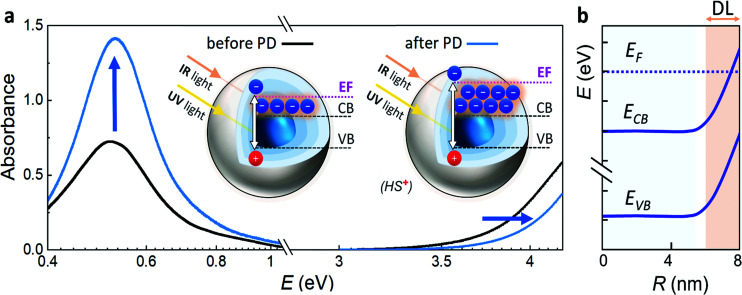
(a) Photodoping effects on the optical spectrum of doped MO NCs. Typical localized surface plasmonic resonance (LSPR) of ITO NCs before (black line) and after (blue line) the photodoping process peaking at around 0.5 eV. With UV exposure the peak position of the plasmon resonance slightly blue shifts and its absorption significantly increases. The Moss–Burstein effect after photodoping results in the blue-shift of the absorption onset in the UV spectral range. Inset shows an illustration of the Fermi energy shift before and after the photodoping process with UV light and the resonant absorption of IR light by the free electrons. (b) Illustration of the electronic structure of a MO NCs with the Fermi level pinning and the consequent formation of an electronically depleted layer (DL).

Electron paramagnetic resonance spectroscopy and theoretical calculations unveiled that the extra electrons introduced *via* photodoping are delocalized.^12,34,53^ Such charges add to the initial carrier density of the doped MO, together resulting in localized surface plasmon resonances (LSPRs) (Fig. 3a – black line).^9,54,55^ These plasmonic resonances are very sensitive to the carrier density, size, shape, or dielectric surrounding and therefore hold as a source of information for the doping processes occurring in MO NCs.^8,9,55–58^ Owing to the proportionality between the LSPR (*ω*_LSPR_^2^, see below) and the free carrier density (*n*_e_), spectroscopic investigation allows monitoring the doping process.^8,10,24,59–62^ In fact, the process of photodoping in doped MO NCs can be tracked in real-time by following the spectral modification of the localized plasmonic resonance. Experiments on ITO NCs with increasing UV exposure time and anaerobic conditions show a blue shift of the LSPR peak, which is also accompanied by a significant increase of the plasmonic response (Fig. 3a).^32^ Furthermore, the introduction of extra charges and the consequent increase in carrier density induces a shift in the absorption edge of MO NCs, which appears as an increase in the optical bandgap due to the filling of the lowest states of the conduction band. This phenomenon is known as the Moss–Burstein effect^63^ (Fig. 3a). The spectroscopic signatures serve additionally as a feedback parameter for the potentiometric titration experiments (*e.g.*, the normalized integrated intensity of the LSPR). The necessary equivalents of titrants to turn the system to its initial conditions correspond to the number of extra electrons stored in the system (as illustrated in Fig. 2b).^32^

Several models established for conventional plasmonic materials have been adapted to describe the optical response of MO NCs.^9,56,64^ A commonly used description relies on Mie scattering theory in the quasi-static approximation with absorption and scattering cross-sections given by:2
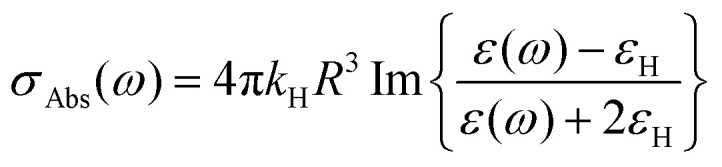
3
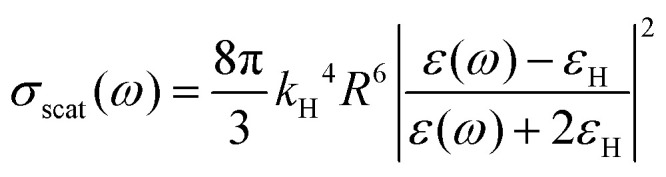
with *R* the radius of MO NCs, 
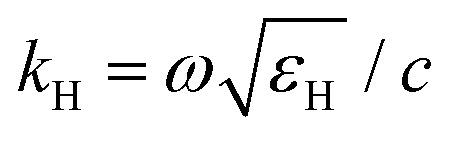
 the wavevector in the host medium of dielectric permittivity *ε*_H_ and *ε*(*ω*) the nanoparticle Drude-like complex dielectric permittivity *ε*(*ω*) = *ε*_∞_ − *ω*_P_^2^/(*ω*^2^ + i*ωΓ*). From eqn (2) and (3), the LSPR frequency *ω*_LSPR_ is provided by the so-called Fröhlich condition, Re(*ε*(*ω*)) = −2*ε*_H_, leading to:4
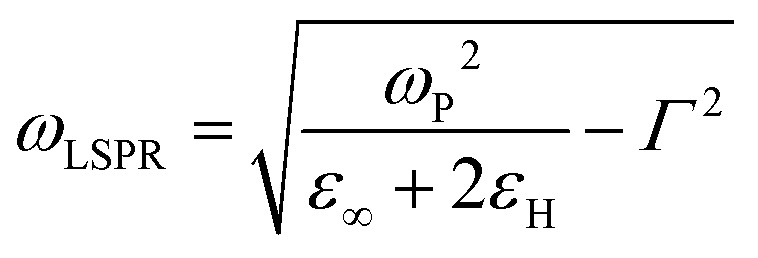
where 
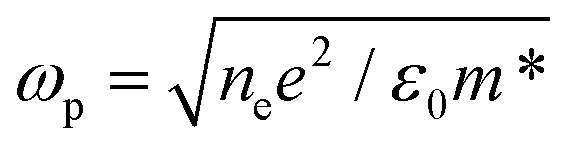
 is the bulk plasma frequency with *n*_e_ the free carrier density, m* is the effective electron mass and *Γ* is a damping parameter.

Other models to characterize the modification of MO NCs consist of the implementation of core–shell structures to describe the post-synthetic doping process.^59,64–66^ Such models were adapted to track the optical changes occurring by introducing extra electrons in an electrochemical charging process. Here, the core represents the doped MO, while the shell describes a carrier depleted MO.^32,59,65,66^ The depletion of carriers close to the surface is a result of surface states, which are located energetically below the Fermi level and the related phenomenon of Fermi level pinning. This induces an upward bending of the conduction and valence band and results in the creation of a layer with heavily suppressed electron density extending over several nanometers into the material (Fig. 3b). It has been reported that the presence of an electronically depleted region affects many applications of MO NCs that rely on conductive films, such as displays or electrochromic windows.^59,65,66^ Recent studies have linked the electrochemical introduction of extra electrons to a decrease of the depletion layer.^65^ Furthermore, Morfa *et al.* reported a conductivity enhancement after UV illumination by a factor greater than two in thin films based on MO nanocrystals,^67^ suggesting that depletion layer modulation plays a key role in the photodoping and energy storage processes as well. This light-induced modulation could be exploited as a last-stage technique to optimize device performances whenever depletion layers are detrimental for the application needed.

While such models support well the experiment,^32^ they might neglect details related to charge transfer or not appropriately consider nanoscale structural effects. The Drude model is derived for bulk materials and assumes a uniform electron distribution, which might not be valid at the nanoscale. Dopant segregation or dopant distribution influence locally the electronic properties of the material particularly in the near-surface region,^59,65^ which becomes prominent at the nanoscale due to the large surface to volume ratio. Additionally, charge balancing cations attached to the nanocrystal surface interact with the capacitive electrons in the system resulting in variations of the NCs’ Fermi levels and, in turn, affecting their chemical reactivity.^40,41^ Carriers introduced by chemical dopants are always connected to a positive lattice site, opposed to those introduced *via* photodoping, and therefore might display a different dielectric response than the capacitive carriers.^68^ This is a very important aspect in applications that rely on precisely determining the number of charge carriers within the nanostructure.^69^ Moreover, in bulk materials, the band edge and Fermi energies are rigorous thermodynamic descriptors.^70^ In nanoparticles, a change of one electron could cause a significant nuclear reorganization of the system due to the small size. This is often related to additional effects, such as ion transfer followed by ion intercalation, surface binding, or movement within the surrounding bilayer, as well as solvent reorganization and vacancy formation.^70^ Energetics related to such nuclear reorganization or cationic coupling are usually not included in Fermi and band energies, while these effects can be significant at the nanoscale.^70^ Therefore, a comprehensive approach to the thermodynamics of charge transfer at the nanoscale should explicitly consider the aforementioned effects, in order to have a more complete understanding of reaction thermochemistry and control of redox reactivity in MO NC photodoping.^65^

## Perspectives

The possibility to combine both light conversion and energy storage in a unique system holds promising perspectives for light-driven energy storage and self-powered nanodevices. Integrated and rechargeable storage devices, employing either a solar cell or a photoelectrode as the light-to-charge conversion unit, represent the current state of the art.^5^ However, energy and voltage matching during the coupling of two separated subunits limit the overall efficiency of these devices. In addition, a rather complex implementation process may also restrict their future application space.^5^ Compact, portable and integrated devices, able to couple together light-harvesting and storage are required. Photodoping in MO NCs holds promise to fill this gap by combining light absorption, charge separation, and accumulation in the same set of materials.^9^ Additionally, MO NCs have the potential to merge solar-powered energy storage with multiple charge transfer capability.

In this context, different energy storage technologies can be described and compared from a technical perspective.^48,71–77^ In recent years two main complementary approaches emerged in the field of energy storage, depending on the primary technological need to address. Specifically, it is possible to design high-performance energy storage systems that can store energy for long periods, such as batteries, or that can supply concentrated power in a short time, such as capacitors. To evaluate their technical performance, several figures of merit are considered such as efficiency, energy capacity, energy density (energy accumulated per unit of volume or mass), power density (speed of energy transfer per unit volume or mass), response time, lifetime in years and cycles, and self-discharge.^72,75,78^ Most common types of batteries include lithium-ion (Li-ion), sodium–sulfur (NaS), nickel–cadmium (NiCd), lead-acid (Pb-acid), lead–carbon batteries, and zebra (Na-NiCl_2_) and flow batteries; while capacitors can be classified as electrostatic capacitors, electrolytic capacitors and supercapacitors. Among these three types, supercapacitors have the highest capacity per unit volume due to the porous structure of the electrode.^78,79^

In a notable example of doped MO NCs, Fe doped ZnO NCs were described as soluble supercapacitors, with capacitance values as high as 233 F cm^3^ and 33 μF cm^−2^, for volumetric and areal capacitances respectively,^17^ rivalling the performance of commercially available supercapacitors.^6,29–33^ In this example, the Fe-doping introduces extra acceptor levels with energy just below the conduction band. After photodoping, charges are stored in these highly localized orbitals and still easily removed as a result of the equilibrium condition between the Fe levels and the conduction band of the MO NCs.^17^ The number of extra charges increases significantly due to Fe doping, proving that doping control is a promising and viable strategy for the enhancement of the storage capacity of MO NCs and represents the first indication for the potential of optimized doped MO NCs as light-driven supercapacitor materials.^17^ Due to the novelty of this approach there are, up to date, only a limited number of proof-of-concept supercapacitors based on MO NCs and, hence, we foresee important breakthroughs through the development of optimized devices in the incoming years.

To reach such goals, the light-induced charge accumulation process now requests its transformation into high-performance and long-lasting solar energy storage solutions.^5^ Research carried out over the past decade has shown that NCs, in general, present many advantages in energy storage in terms of high packing density, high surface-to-volume ratio, and short diffusion pathways, providing opportunities for simultaneously achieving high energy and power density.^80^ This versatility of NCs combined with the benefit of light-driven charge accumulation can open avenues to novel renewable energy storage designs.

**Fig. 4:**
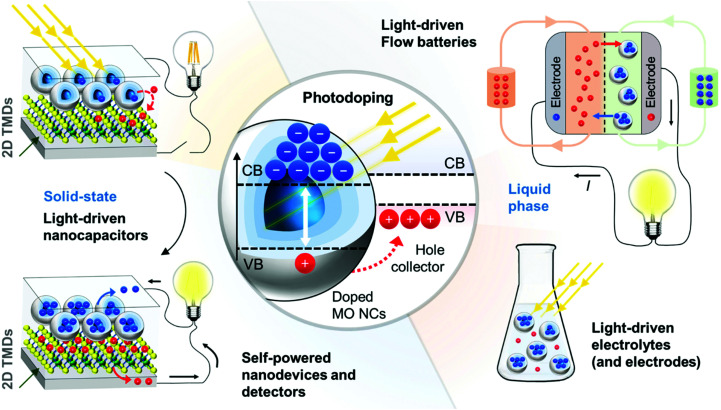
Possible architectures for light-powered energy storage solutions and light-driven nanodevices based on the photodoping of doped MO nanocrystals. On the left side: All-solid-state light-driven capacitor based on the coupling between MO NCs and a monolayer 2D transition metal dichalcogenides (TMDs). Center: Illustration of photodoping in the presence of a hole collector with proper band alignment. Light triggers the photodoping process and photo-generated charges are transferred to the hole collector. On the right side: Possible application of MO NCs in solution illustrating a light-driven flow cell battery architecture (on top) and light-charged MO NC-based electrolytes.

A promising route to exploit photodoping for solar energy storage is by developing light-driven electrodes (Fig. 4). An important factor for efficient storage functionality is the electrodes’ design. High specific capacitance becomes achievable when enhancing the electrode/electrolyte interface area. Together with small charge layer separation of atomic dimensions, high energy densities are expected.^81,82^ In the state-of-the-art supercapacitor architectures, the predominant trend is to implement micro-supercapacitors with three-dimensional architecture.^3^ The latter essentially involves anodes and cathodes with exposed surfaces in three dimensions rather than the flat surface of conventional thin films, exploiting many nanostructured morphologies, such as nanocrystals, nanowires, nanotubes, nanosheets, and nanowalls.^3^ Such principles can be directly translated to MO NC-based electrodes to enhance specific capacitances after light absorption. Additional requirements to the employed MO nanostructures are related to high ionic adsorption capacity at the solid–liquid interface, fast and reversible surface redox reactions, and fast charge transfer as well as high conductivity, highlighting the requirement for further dedicated material design.^3^

A notable advantage of photodoped MO NCs lies in the opportunity to exploit multi-charge transfer steps. From 
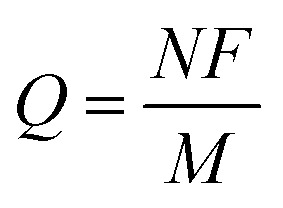
, it is clear that the theoretical specific capacity (*Q*) directly relates to the number of electrons *N* involved in the reaction, the Faraday constant (*F*), and low molecular weight (*M*). Such multi-charge transfer reactions are considered key tools for achieving high energy density in batteries. This highlights MO NCs with multiple charge transfer capability as high energy density components in the next-generation of energy storage devices. Current multi-charge transfer components in energy storage suffer stability issues^83^ due to the formation of highly reactive species during the charge transfer.^84^ Superior stability through the delocalization of the stored charges^64^ may offer new effective solutions in particular when implemented in devices in remote places, which require technologies with a long service-life.

Photodoping of MO NCs in solution becomes interesting when considering functional light-driven nanoinks, in which the dispersed colloids can store multiple charges per nano-unit and act simultaneously as the energy-carrying electrolyte (Fig. 4, right panel).^80^ A visionary route is the development of solar rechargeable flow-cell systems by coupling the light-driven MO nano-inks to well-established device compartments already used in flow batteries.^85^ Systems, in which the charge-bearing electrolyte is spatially separated from the electrode, as in flow cell devices, deliver benefits to individually tune the energy and power density of the system, highlighting all together the benefit of implementing MO NCs in novel energy storage solutions (Fig. 4, right panel).

A very important issue in current photodoping approaches, is the loss of the photoexcited hole in the photodoping process, which is sacrificed by reacting with the hole scavengers in solution. In this way, only half of the carriers contribute to solar energy conversion and storage. For a fully functional device, however, it is important to replace the currently employed sacrificial hole scavengers with appropriate hole collectors that can fully participate in the charge storage process. Molecules or nanoparticles of materials that are capable of accepting multiple holes from the photodoped MO NCs and redeliver them to appropriate electrodes are required. As a first example, monolayer two-dimensional transition metal dichalcogenides (2D TMDs) were employed as all-solid-state hole-acceptors, accepting permanently multiple photo-generated holes from ITO NCs.^18^ This work displays the successful charge separation and stable accumulation of multiple electrons and holes in separate units after photodoping, where the NC film represents the negative electrode, and the 2D TMD the positive one. The nanometer separation of the two separated plates when assuming a capacitor-like system composed of two plates of opposite charges highlights promising capacitance values of femto Farads (corresponding to areal capacitances of ∼μF cm^−2^). Energy and power densities in the range of μJ cm^−2^ and μW cm^−2^ are foreseen.^18^ To complete the fully functional, light-driven nano-capacitor device however fully functional charge extraction electrodes are required (Fig. 4, left panel).

In this example, the hypothesized cell voltage was derived from the energetic difference between the conduction band minimum and the valence band maximum of the electron and hole acceptor, respectively, resulting in an expected cell voltage of around ∼1.2 V.^18^ While this serves only as a first estimate, it raises important questions regarding the expected properties of proper hole collectors. The band offset will have an important impact on the output potential in the solar-energy storage device for a given MO-hole collector pair and their band alignment represents a key requirement to enable efficient light-driven charge separation (Fig. 4, central panel).

For competitive solar conversion efficiency, the absorption of the involved materials needs to be extended and red-shifted from predominantly UV to the visible part of the spectrum. Recent works described the capacitive properties of InN NCs.^86,87^ These have a significantly lower bandgap in the visible/near-infrared regimes. Photodoping of such NCs would represent an important step towards storing a larger potential of solar energy. Also the participation of an active hole collector in light absorption and electron donation would beneficially contribute to the overall efficiency of the devices. Designed in an appropriate way this might enable the extension of light absorption to the red spectral range. Therefore, additional materials research must focus on the development of appropriate hole collectors with multiple charge storage capability. The first examples highlighted that 2D monolayers are an interesting platform,^18^ while the full library of available materials still requires thorough investigation. This displays the importance of materials’ research in the study of MO NCs for light-driven energy storage. Another key point is the implementation of environmentally benign and non-toxic elements. In fact, the study of photodoping was so far limited to few metal oxides. The exploitation of the richness of chemical synthesis techniques will expand the list of materials to low-cost, environmentally friendly, and abundant materials.

Apart from energy-related applications, doped MO NCs appear interesting as active elements for other light-driven optoelectronic nanodevices as well. The possibility to modulate the carrier density of doped semiconductors *via* photodoping could be exploited to design transistors in which the gate input is replaced with light, or even developing all-optical-input.^88,89^ In fact, in a recent work MO NCs were employed as all-optical light-driven charge injection tools to inject multiple electrons into 2D TMDs, in analogy to 2D material gating.^89^

This together displays that MO NCs photodoping opens horizons for novel application spaces as active elements in self-powered nano-electronics. Moreover, light-driven MO NCs in proper device architectures display a promising and viable strategy for novel solar-powered energy storage units. With the focused research from the materials to the devices, MO NCs have the potential to contribute to a future sustainable and zero-emission energy landscape by exploiting fundamentally new concepts of solar energy conversion and storage.

## Conflicts of interest

There are no conflicts to declare.
